# BERT’s sentiment score for portfolio optimization: a fine-tuned view in Black and Litterman model

**DOI:** 10.1007/s00521-022-07403-1

**Published:** 2022-05-30

**Authors:** Francesco Colasanto, Luca Grilli, Domenico Santoro, Giovanni Villani

**Affiliations:** 1 Department of Mathematics, University of Florence, Florence, Italy; 2 Department of Economics, Management and Territory, University of Foggia, Foggia, Italy; 3 Department of Economics and Finance, University of Bari, Bari, Italy

**Keywords:** Sentiment analysis, BERT, Black and Litterman model, Portfolio optimization, 60G50, 62M20, 62M45, 91B70, 91B84

## Abstract

In financial markets, sentiment analysis on natural language sentences can improve forecasting. Many investors rely on information extracted from newspapers or their feelings. Therefore, this information is expressed in their language. Sentiment analysis models classify sentences (or entire texts) with their polarity (positive, negative, or neutral) and derive a sentiment score. In this paper, we use this sentiment (polarity) score to improve the forecasting of stocks and use it as a new “view” in the Black and Litterman model. This score is related to various events (both positive and negative) that have affected some stocks. The sentences used to determine the scores are taken from articles published in *Financial Times* (an international financial newspaper). To improve the forecast using this average sentiment score, we use a Monte Carlo method to generate a series of possible paths for several trading hours after the article was published to discretize (or approximate) the Wiener measure, which is applied to the paths and returning an exact price as results. Finally, we use the price determined in this way to calculate a yield to be used as views in a new type of “dynamic” portfolio optimization, based on hourly prices. We compare the results by applying the views obtained, disregarding the sentiment and leaving the initial portfolio unchanged.

## Introduction

A large amount of information extracted from the markets today allows investors to adjust their strategies quickly. However, some types of information (e.g., those expressed in natural language) are difficult to match at the price of a stock. For example, a sentence expressed by a prominent figure of a company, or a comment expressed in an influential newspaper or on a particular social networking site, could be an important indication for the investor who is undecided on which strategy to take.

Given the time series of a stock, a current problem for investors is how news expressed in natural language can be integrated into subsequent changes in that stock’s price (or yield). In this paper, our goal is to transform that variation opinion into a numerical value and propose a heuristic that can integrate that value into the time series to improve prediction in the very short term. We use a Natural Language Processing (NLP) model called FinBERT, focused on the economic/financial sector, which can generate a numerical value of a sentence in natural language. This value will allow us to improve the trajectory of the time series and, consequently, improve the allocation of assets in a financial portfolio effectively. Unlike many models where sentiment is used to understand whether prices in the stock markets will rise or fall, we use sentiment analysis to determine a score for improving the prediction of a stock’s future price, exploiting the properties of Brownian motion (the stochastic process that describes the dynamics of stocks). Then, this score is used to determine a return through a simulation process. In addition, this yield will be used to input Black and Litterman’s [1] model (BL) to adjust the portfolio composition (trying to avoid losses). The main novelty that this paper intends to introduce is a method to “quantify” the intensity of news that will influence the choice of stocks in a financial portfolio. Usually, the Black–Litterman model allows the insertion of a view (a numerical parameter that describes how the price of a stock in the portfolio could change). The main problem is that the manager/investor is forced to enter a single value, which is the synthesis of his feelings (possibly born from the news perceived by the markets), a value that could easily be too unbalanced in positive or negative, precisely because of the human rationality. This methodology aims to assist the investor in the “quantification” of his feeling and optimal choice of that single numerical value.

Regarding investor behavior, the most established theory is that of Tversky and Kahneman [2], defined as Cumulative Prospect Theory (CPT), which considers the choices individuals make in practice (by improving the theory of expected utility (EUT)). In the case of portfolio choice, many theories govern investor behavior, such as Shefrin and Statman [3] , according to which choices are based on the potential/aspiration (SP/A) theory. He and Zhou [4] develop a model of choice based on hope, fear, and aspiration; again, Bi et al. [5] , in which the cash flows are a tool for determining the probabilities of allocation weights. We can start from the results of Harris and Mazibas [6], according to which behavioral investors perform better than naive or rational investors, in order to consider our methodology consistent with behavioral theory; or the Barberis et al. [7] theory, in which investors believe that the past performance of a financial asset is a good proxy for its future return. We can exploit this investor consideration and make it part of the methodology, even as a starting point for the Black–Litterman model.

### Proposal approach

The main difference between the econometric or neural approaches and the one proposed in this paper is related to the use of the sentiment score. Generally, the econometric approach considers the stock price time series as a process and studies its characteristics (primarily through statistical tools). In our methodology, on the other hand, we propose an approach based more on measure theory. We try to identify a region (which will later be identified as Borelian) within which the stock price will end, directed by the polarity score. Unfortunately, this score (being a single numerical value) does not lend itself to use through time series analysis tools nor to be enhanced for more modern approaches such as Machine/Deep Learning. For example, making this score a *data sets feature* would be a problem, especially in the training phase, since its determination depends on the presence of sentences (in natural language) for each stock, thus making us run into a possible lack of data.

The application of the sentiment score to the Black–Litterman model is linked to the fact that this represents the state-of-the-art for portfolio managers, particularly its diffusion and ease of use in applications. The advantage is introducing only a change in the choice of views while leaving the friendliness of that model unchanged instead of proposing an entirely new methodology that may not be completely clear to the user.

### Paper’s organization

The paper structure is: in the following Sect. 2, we analyze the reference literature and the primary BERT model considered; in Sect. 3 the previous architecture (FinBERT) will be applied to a series of articles concerning some stocks to determine a score, which will be used to forecast the future price using the Monte Carlo method. Section 4 highlights the results obtained by applying the previous methodology and comparing the final composition of the portfolio in the Black and Litterman model with different types of views; finally, in Sect. 5, some conclusions are drawn. Furthermore, in “Appendix A”, there is a comparison between the results obtained with our heuristics and some of the leading models known in the literature.

## Related work

In finance, one of the main objectives has always been to determine the future price of an instrument. The dynamic study of stock in the financial markets, represented through time series, shows that many factors influence its performance. The most common practice for making forecasts is to focus on logarithmic returns to link financial theory with statistical analysis. One of the first ideas introduced by Fama [8] in the *Efficient Market Hypothesis* (EMH) theory holds that it would not be helpful to consider historical data in analyses to make predictions since current prices are independent of historical ones. The historical approach is based on the study of the trend, as shown by Fama and French [9] that uses expected returns to generate temporary price components, or Perasan and Timmermann [10] who consider the power of various macroeconomic factors on the markets. Time-series forecasting, from an econometric point of view, has developed through the transformations proposed by Box and Jenkins [11], which state that the use of a power transformation leads to obtaining an adequate *Autoregressive Moving Average* (ARMA) model [12]. Several evolutions followed this pattern. For example, Coffie [13] proposed a *Glosten Jagannathan and Runkle* (GJR) version of the *Generalized Autoregressive Conditional Heteroscedasticity* (GARCH) model to capture the leverage effect on the stock market. Siouris and Karagrigoriou [14] introduce a correction that considers the asset prices to improve the Value at Risk (VaR) forecast. Bucevska [15] highlights how, among the different GARCH models, the most suitable for estimating the volatility in the equity markets is the EGARCH. Alternatively, Mantalos et al. [16] study the skewness of the time series by proposing a series of tests for the normality of the data by applying the *Normalizing and Variance-Stabilizing Transformation* (NoVas). In particular, they provided a background for skewness test on series with ARCH/GARCH effect, highlighting how this methodology introduces appropriate tests for skewness/kurtosis and behaves better than traditional techniques for prediction. In markets, as well as in the economy more generally, the question of information is essential. The determination of a future price is significantly influenced by the presence or absence of information that an investor has about a particular instrument. In the financial markets, where the difference in information is increasingly evident, these subjects base their choices on history [17]. Moreover, as Keynes [18] defined, financial markets are characterized by the *euphoria* that makes investors not attentive to long-term returns, focusing only on random short-term ones. This condition implies that investors ignore the events that happen and have too much confidence in themselves [19] (Simon [20] defines the limit on the ability to process information as *“bounded rationality”*).

In the present paper, we try to exploit the information obtainable from the financial markets (e.g., through newspapers) to improve the forecast of the price trend of a financial instrument. Thanks to the spread of social networks, many methods are present in literature to exploit the diffusion of information in the stock markets, especially in recent years. For example, Tetlock [21] quantitatively measures the interactions between the performance of stocks and media content, highlighting how a pessimism spread by the media predicts downward situations in prices or volumes. Schmeling [22] studies how investor sentiment affects stocks returns, demonstrating how the impact of sentiment on returns is more significant for countries with less integrity in the markets. Joseph et al. [23] examine a sentiment-based method for predicting abnormal stocks returns. Bollen et al. [24] study the correlation between the sentiment spread among investors through Tweets and the value of stocks indices. Preis et al. [25] analyze the link between research carried out in financial fields (Google query) as a tool for predicting signals in market movements. Cosimato et al. [26] study how a large amount of information on social media helps predict outcomes of real-world. Finally, Guo [27] uses the Thermal Optimal Path method to show the relationships between sentiment and financial markets, highlighting how sentiment is useful when excellent investor attention is on the stock.

As for modern approaches, there have been innovations in stock price prediction and portfolio management. For example, Refenes et al. [28] used a neural network system to forecast the exchange rates via a feedforward network. Sharda and Patil [29] compared the predictions obtained using neural networks and the Box–Jenkins model. This comparison verified that neural networks performed better than expected for time series with a long memory. Andrawis et al. [30] combined the forecasts obtained via different time aggregation, and Adeodato et al. [31] proposed a methodology based on an ensemble of Multilayer Perceptron networks (MLPs) to achieve robust time-series forecasting. Namdari and Li [32] on the other hand, use an MLP with three hidden layers to develop a predictive model of the stock price that considers the results of technical and fundamental analysis; extending the work (Namdari and Durrani [33]) in a separation between technical and fundamental analysis, demonstrating how the hybrid model is more predictive than single analyzes. With more complex architectures, Kim et al. [34] have proposed a Hierarchical Attention network for stock prediction (HATS), which uses relational data for stock market prediction. HATS can aggregate information and add it to the nodal representation of each company, demonstrating how it outperforms all existing methods since it automatically selects information. Alternatively, Fischer and Krauss [35] develop a Long-Short Term Memory (LSTM) network to predict the directional movements of the *S&P500*, highlighting how this architecture outperforms methods such as Random Forest, Deep Neural Network (DNN), and Logistic Regression Classifier.

From the side of portfolio optimization, since these are multi-objective problems, modern artificial intelligence techniques (such as neural networks or genetic algorithms) have allowed an adequate resolution. Leow et al. [36] use a BERT-based model to capture information on market conditions and a Genetic Algorithm to maximize returns and minimize portfolio volatility. Sawhney et al. [37] start from the difficulties of training neural networks for forecasting in a financial context characterized by different types of information. They propose a learning approach using entropy-based heuristics. Pal et al. [38] propose a method to improve the choice of stocks to be incorporated into the portfolio based on a Non-dominated Sorting based Genetic Algorithm (NSGA-II) for the choice of portfolios and a single objective Genetic Algorithm based on the Markowitz model to identify the stocks inside. Wang et al. [39] , using fuzzy set theory, introduce the Sharpe index in fuzzy environments and propose a fuzzy Value-at-Risk ratio, creating a multi-objective model to assess the joint impact on portfolio selection. Ban et al. [40] introduce Performance-Based Regularization (PBR) to bound the variances of estimated risk and return for problems in portfolio optimization. Aboussallah and Lee [41] use a Reinforcement Learning approach (in particular Recurrent Reinforcement Learning (RRL)) to build an optimal real-time portfolio, capable of capturing market conditions and rebalancing the portfolio accordingly. Liang et al. [42] use Reinforcement Learning (RL) agents such as Deep Deterministic Policy Gradient (DDPG), Proximal Policy Optimization (PPO), and Policy Gradient (PG) to test different combinations of parameter settings, feature selection, and data preparation. Alternatively, Koratamaddi et al. [43] propose an RL system for automatic trading that considers the market sentiment of a portfolio obtained through social media, thus considering the investors’ intentions represented by the sentiment.

### Sentiment analysis with BERT

One of the tasks of Natural Language Processing (NLP) is represented by the sentiment analysis [44], or the analysis of human opinions through text expressions. The main objectives are, on the basis of different levels of granularity (document, sentence, aspect): obtain classifications based on polarity (positive/negative/neutral expression), subject classification (distinguish whether an expression is subjective or objective) and irony detection (check if an expression is ironic or not). There are many potential applications of sentiment analysis, especially thanks to the advent of social networks and it is use in fields of consumer products, health care, financial services (stock market prediction), and political elections.

Recent developments in NLP have made it possible to combine the best features of models such as Embeddings from Language Models (ELMo) and Generative Pre-trained Transformer (GPT), obtaining a model that allows the encoding of context in a bidirectionally usable manner in various NLP tasks. Bidirectional Encoder Representations from Transformers (BERT [45]) is a new type of architecture based on an encoder–decoder network that uses the transformer-based architectures [46]. This model consists of a set of transformer encoders which perform two fundamental tasks: it defines the Language Modeling (MLM) by randomly masking 15% of the tokens (pieces of a sentence generated through the tokenization process) present in the corpora and allows the Next Sequence Prediction (NSP), that is, it predicts if two sentences follow each other. There are two main versions of BERT, BERT-base and BERT-large, both of which are trained on BookCorpus and Wikipedia English for over 3500M words. This model is also used for the sentiment classification task, in particular since many versions trained on corpora of different languages have been created.

Aract [47] used BERT for sentiment classification and regression focusing on the financial world, *FinBERT*. In particular, it’s training was carried out through three datasets: (i) the pre-training was carried out through *TRC2-financial*, filtering the *TRC2* corpus based on financial keywords (obtaining a dataset consisting of over 29M words and 400 k phrases); (ii) *FinancialPhraseBank* was used as the main sentiment analysis dataset [48] and (iii) *FiQA Sentiment*, a dataset created for the $WWW'18$ Conference challenge. This model, despite failing in some cases (as demonstrated in cases where it has difficulty in distinguishing phrases generally used in the business environment from positive ones), turns out to be the best compared to other state-of-the art models and provides great decision support for investors. It was implemented by the authors [47] with a dropout probability of $p = 0.1$, warm-up proportion of 0.2, maximum sequence length of 64 tokens, a learning rate of 2e-5, and a mini-batch size of 64, while for discriminative fine-tuning, the discrimination rate was setting as 0.85. Furthermore, after 10-fold cross-validation, FinBERT achieves a *Loss* of 0.37, with an *Accuracy* of 0.86 and an *F1-score* of 0.84.

## Methodology

FinBERT, as described above, allows us to determine an average sentiment score based on a series of sentences to be analyzed. This means we can obtain an average sentiment score $\gamma \in [-1, 1]$, for negative polarity $\gamma \in [-1, 0)$, while for positive polarity $\gamma \in (0, 1]$. The goal is to use this particular score to allow the investor to improve decision-making on a certain instrument in a short period. In particular, we will focus on forecasting the price of some stocks following news (positive and negative) that could significantly influence investor choice.

### Portfolio optimization

Portfolio managers generally own stocks in several companies to minimize risk (in collections called financial portfolios). We know from economic theory and empirical evidence that the optimal number of stocks in each portfolio is between 10 and 14. This number allows for minimizing the riskiness of individual stocks by balancing any losses with the gains from the others in the portfolio. Suppose to build a portfolio by randomly choosing stocks from the *NASDAQ-100* (a well-known equity index that includes the major non-financial companies, among the most famous globally). In particular, the stocks will be chosen to diversify the companies’ sectors. For example, we can select the $N=10$ stocks listed in Table 1.

**Table 1:** List of stocks in the example portfolio

Company name	Stock code
Apple Inc.	AAPL
Amazon.com Inc.	AMZN
Microsoft Inc.	MSFT
Broadcom Inc.	AVGO
PepsiCo Inc.	PEP
Adobe Inc.	ADBE
Mondelez Int. Inc.	MDLZ
AstraZeneca PLC ADR	AZN
T-Mobile US Inc.	TMUS
Netflix Inc.	NFLX

The managers determine for each total sum *X* to be invested, the percentages allocated to each stock that composes it ($x_k$, s.t. $\sum _{k=1}^N x_k = X$). In particular, these $x_k$ quantities are updated on average every 2 weeks to consider the events that have hit the markets. Let us assume that we are at the end of January 2021 and must adjust the amounts of money allocated to each stock. In this phase, the sentiment score returned by FinBERT comes into play, which allows us to have a judgment (sentiment value) on what happened recently based on the news expressed, for example, in articles in financial newspapers. All the information used for sentiment analysis was extracted from *Financial Times*, an international financial newspaper. Because we are assuming that the investor using this model does not have hidden information about the stock’s performance, we have considered some excerpts from these articles. For selecting the sentence in an article, we take into account all sentences except those whose polarity is repeated the same in succession for more than two sentences. For example, denoting with $+$ positive polarity and with − negative polarity, a correct sequence is of type $\{+, +, -, +, -, -, \dots \}$, while in the case of a succession $\{-, -, -, +, +, -, \dots \}$, we would eliminate a negative sentence in the top three.

At the end of January 2021, the articles to consider will refer to the companies we have in our portfolio. However, which were published not many days before the end of the month: this selection will allow us to avoid potential losses until the next rebalancing. Based on this, the articles considered are:

- **Microsoft**, in an article published on 1/27/2021.^1^ The main topic concerns the increase in company turnover following the boom in PC sales, despite the pandemic situation (**positive** score);
- **Mondelez**, in an article published on 1/28/2021.^2^ The main topic concerns a possible violation of the competitive behavior of the chocolate producer, obtained by limiting cross-border trade (**negative** score);
- **Apple**, in an article published on 1/28/2021.^3^ The main topic concerns the increase in company turnover in the last period, despite the scarcity of components (**positive** score);
- **Amazon**, in an article published on 1/29/2021.^4^ The main topic concerns the use by US law enforcement agencies of Amazon security systems that could violate the privacy (**negative/neutral** score);
- **AstraZeneca**, in an article published on 1/29/2021.^5^ The main topic concerns publishing of the pharmaceutical company’s contract on the Covid-19 vaccine (**neutral/negative** score).

Once the article sentiment score has been determined as the average difference between the positive and negative score for each sentence, the challenge is to connect it (the so-called $\gamma$) to the changes that could occur in the stock’s price. However, using it directly to predict a future price simply by adding or subtracting (based on the polarity obtained by FinBERT) it from the current price would be an error. First, the score only expresses the intensity of a specific polarity based on sentences used; second, the forecast time is not specified. For this reason, to solve the problem, we use the following heuristic: starting from the definition of the space in which the stock price can move, we apply a new measure that allows the discretization (therefore the counting) and the consequent creation of a range of variation. In this way, the future price can be determined in the new range. From a portfolio perspective, we can use the predicted price several days in the future to adjust the quantities $x_k$ to invest in each stock, all based on sentences expressed in natural language.

#### Italian case

We can also test FinBERT on newspaper articles not initially written in English. For example, we can consider some stocks belonging to the FTSEMIB (the market index containing the essential Italian stocks) and verify, over a certain period, what was the most important news for that company. The information is taken from *MF Milano Finanza*, an Italian economic-financial newspaper. The translation of the sentences from Italian was carried out by an English native speaker expert in the domain. In this case, however, since we are only interested in verifying the effectiveness of the forecasting method for the price (and not in optimizing a portfolio), we choose the sentences within each article as in the previous case. However, we do not give much importance to the fact that this news refers to days not so close (the reference period in this example was November 2020). Therefore, the stocks considered (all listed on *Borsa Italiana*) are:

- **Brembo**^6^ (BRE.MI), in an article published on 27/11/2020. The main topic concerns the lowering of the company’s rating due to the strong pressures caused by the pandemic and after reaching the target price set by analysts (**negative**);
- **Unicredit S.p.A.**^7^ (UCG.MI), in an article from 29/11/2020. The main topic concerns the failure to reapply the company’s CEO to his current position and some reasons that may have led him to take this type of decision (**negative**);
- **EssilorLuxottica S.A.**^8^ (EL.MI), in an article on 27/11/2020. The main topic concerns the possible acquisition, by the eyewear giant, of a Canadian company very widespread in North American territory (**positive**);
- **UnipolSai Assicurazioni**^9^ (US.MI), in an article from 13/11/2020. The main topic concerns the analysis of the insurance company management in the quarters Q1-Q3 of 2020 (**positive**).

The sentiment scores, in this case, will only be used as a prediction accuracy test but not for portfolio optimization.

### Formulation of the problem

First of all, we provide some preliminary definitions of probability theory and stochastic calculus.

#### Definition 1

($$\sigma -algebra$$ of $$\mathbb {R}$$)

$$\begin{aligned} \mathcal {M} \; is \; a \; \sigma -algebra \; of \; \mathbb {R} \iff \end{aligned}$$

- $$\mathbb {R}, \emptyset \in \mathcal {M}$$
- $$\mathbb {R} \setminus A \in \mathcal {M} \quad \forall A \in \mathcal {M}$$
- $$\bigcup _{n=0}^{\infty } A_n \in \mathcal {M} \quad \forall \{A_n\}_{n\in \mathbb {N}}$$ sequence of $$\mathcal {M}$$.

#### Definition 2

(Borel $\sigma$-algebra of $\mathbb {R}$) We define $\mathcal {B}(\mathbb {R})$ the $\sigma -$algebra of Borelians of $\mathbb {R}$ as the smallest (with respect to the inclusion relation $\subseteq$) $\sigma -$algebra of $\mathbb {R}$ which contains all ranges of the type $(-\infty ;a), (a;+\infty )$ for all $a\in \mathbb {R}$.

#### Definition 3

(Borel Set of $\mathbb {R}$)

$$\begin{aligned} A\subseteq \mathbb {R} \; is \; a \; Borel \; set \iff A\in \mathcal {B}(\mathbb {R}). \end{aligned}$$

Intuitively, the Borel Sets represent all the possible prices that can be obtained in a certain moment *t*. In this way, we used the sentiment score to determine a specific measure on a Borel set $A \subset \mathbb {R}$ in which some Brownian motions fall. We know that a stock’s price has dynamics described by the following *stochastic differential equation* (SDE) [49]:

1 $$\begin{aligned} dX_t = b(t, X_t)dt + \sigma (t, X_t)dB_t, \end{aligned}$$

where *b* and $\sigma$ are given functions and $B_t$ is a Wiener process. A stochastic differential equation describes the motion of a particular non-deterministic dynamic system (in our case, the stock price). In particular, its solutions will not be real functions but the stochastic processes $X_t$, i.e., all the possible trends that occur created because of any disturbance modeled by the Wiener $B_t$ process. The process $X_t$ (denoted by $S_t$) is called *Geometric Brownian motion* (GBM, since the solution is in the form $X_t = X_0\cdot e^{\mu t + \alpha B_t}$) and, almost always, the SDE representing the price dynamics [50] is:

2 $$\begin{aligned} dS_t = \mu S_tdt + \sigma S_t dB_t, \end{aligned}$$

in which $\mu$ represents the *drift* and $\sigma$ the *volatility*.

We want to put a reasonable measure (lately called Wiener measure) on the space of all possible paths of $S_t$ with a prescribed initial value $S_0$ in $t_0$. The classical framework to develop this tool is the probability theory on generalized product probability spaces and so we fix some notation about that.

Let $\Lambda$ be a nonempty set and $\{X_{\lambda }\}_{\lambda \in \Lambda }$ a family of sets indexed by $\Lambda$, then we can define the generalized product space of $\{X_{\lambda }\}_{\lambda \in \Lambda }$ in the following way:

$$\begin{aligned} \prod _{\lambda \in \Lambda }X_{\lambda }:=\{g:\Lambda \rightarrow \cup _{\lambda \in \Lambda }X_{\lambda } \; \; \; s.t \; \; g(\lambda )\in X_{\lambda } \; \; \; \forall \lambda \in \Lambda \}. \end{aligned}$$

Moreover, for all $\emptyset \ne F\subseteq \Lambda$ we define the canonical projection $P_F:\prod _{\lambda \in \Lambda }X_{\lambda } \rightarrow \prod _{\lambda \in F }X_{\lambda }$ in this way:

$$\begin{aligned} P_F(g):=g_{\vert F} \qquad \forall g\in \prod _{\lambda \in \Lambda }X_{\lambda }. \end{aligned}$$

Lastly, we explaying the useful tool of push-forward measure: let $(X, \mathcal {M}, \mu )$ be a measure space, $(Y, \mathcal {N})$ a measurable space and $f:X \rightarrow Y$ a measurable function, then we put on *Y* a $\mathcal {N}-$measure defined in this way:

$$\begin{aligned} f_{\sharp }\mu (A):=\mu (f^{-1}(H)) \qquad \forall H\in \mathcal {N}. \end{aligned}$$

Let $\Lambda$ be a nonempty set and $\{(X_{\lambda },\mathcal {M}_{\lambda })\}_{\lambda \in \Lambda }$ a family of measurable spaces indexed by $\Lambda$. On the set $\prod _{\lambda \in \Lambda }X_{\lambda }$ (the generalized product space of $X_{\lambda }$), we put the $\sigma -$algebra $\bigotimes _{\lambda \in \Lambda }\mathcal {M}_{\alpha }$ that is the smallest $\sigma -$algebra on $\prod _{\lambda \in \Lambda }X_{\lambda }$ that makes the canonical projection $P_{\{\lambda \}}:\prod _{\lambda \in \Lambda }X_{\lambda } \rightarrow X_{\lambda }$ measurable for all $\lambda \in \Lambda$. In this particular setting, we are interested to the case $\Lambda =[t_0,T]$, $X_{\lambda }=\mathbb {R}$ and $\mathcal {M}_{\lambda }=\mathcal {B}(\mathbb {R})$ (the $\sigma -$algebra of real Borel sets) for all $\lambda \in [t_0,T]$ and so we use the more comfortable notation $\mathbb {R}^{[t_0,T]}$ instead of $\prod _{\lambda \in \Lambda }X_{\lambda }$. Thus, we give the statement of Kolmogorov’s Extension Theorem that is crucial on setting up the Wiener measure on $\mathbb {R}^{[t_0,T]}$:

#### Theorem 1

(Basic Version of Kolmogorov’s Extension Theorem)

*Let* $\Lambda$* be a nonempty set and* $S_{\lambda }$* a Stochastic Process with finite-dimensional distributions* $\mathbb {P}_{F}$* on* $\mathbb {R}^F$* for all* $\emptyset \ne F\subseteq \Lambda$* finite set, then there exists a unique* $\bigotimes _{\lambda \in \Lambda } \mathcal {B}(\mathbb {R})$ -*measure* $\mathbb {P} ^{W_S}$* on* $\mathbb {R}^{\Lambda }$* such that for all* $\emptyset \ne F \subseteq \Lambda$* finite set we have that*

$$\begin{aligned} {P_{F}}_{\sharp }\mathbb {P}^{W_S}=\mathbb {P}_{F} .\end{aligned}$$

For simplicity, from now on we take $t_0=0$, $T=1$ and $S_t$ a Brownian Motion. Using the property of the Wiener Measure about the finite-dimensional distributions, the definition of Brownian Motion and the density of multidimensional Gaussian distribution, we obtain the Wiener Theorem that gives an operative point of view of the Wiener Measure.

#### Theorem 2

(Operative Wiener Measure) *Let* $n\in \mathbb {N} \setminus {\{0\}}$, $0\le t_1< ... < t_n \le 1$, $H \in \mathbb {B}(\mathbb {R}^n)$* and* $S_t$* a Brownian Motion then we have that*

$$\begin{aligned} \mathbb {P}^{W_S}(Q)&=\frac{1}{(2\pi )^{m/2}\sqrt{t_1(t_2 - t_1) \cdot ... \cdot (t_n - t_{n-1})}} \times \\ &\qquad \int _{H} e^{-\frac{x^2_1}{2t_1} + ... - \frac{(x_n - x_{n-1})^2}{2(t_n - t_{n-1})}} dx, \end{aligned}$$

*where* $$Q=\{g\in \mathbb {R}^{[0,1]} \; \vert \; (g(t_1),..,g(t_n)) \in H) \}$$.

The critical element that allows us to discretize (or somewhat approximate) the Wiener measure is the use of *Monte Carlo* simulations. The $\mathbb {P}^W$ measure is applied to the paths’ space generated by the simulations and sets how the polarity can return an exact result. Using the Monte Carlo method allows us to determine a maximum and a minimum value of the paths generated, to which the score is applied as a *weighted average* of the extremes (in which one of the two is the initial price $S_0$).

Analytically, we considered the starting price $S_0$ (for the simulation of the price paths) as the last price recorded on the market in the session preceding the date of publication of the newspaper article, extending it for 45 trading hours (five days). The final price $S_T$ (which, as defined above, discretizes the application of the Wiener measure) is obtained using the sentiment score as an indication of the *percentile* on the interval between $S_0$ and the **maximum** (denoted by $S_{MC}^+$) among the paths generated with the Monte Carlo method at last time *T*, if the polarity is positive; or in that between $S_0$ and **minimum** (denoted by $S_{MC}^-$), if the polarity is negative. The following formula is used to determine the percentiles in the presented case:

3 $$\begin{aligned} S_T ={\left\{ \begin{array}{ll} S_0 + [(S_{MC}^+ - S_0) \cdot \gamma ] &{}\text{ if }\, \gamma \in (0, 1]\\ S_0 - [(S_0 - S_{MC}^-) \cdot \gamma ] &{}\text{ if }\,\gamma \in [-1, 0) \end{array}\right. }, \end{aligned}$$

This method allows us to determine a future price of stocks based on the polarity and sentiment score. At this point we can consider a portfolio of assets to be dynamically “adjusted” through the financial news acquired from newspapers. To do this, we will use the Black–Litterman [1] model, which combines the market equilibrium with the investor’s views to obtain the optimal portfolio composition. Normally, these views can be of a relative or absolute type (relative if comparing the performance of one asset with another; absolute if comparing the performance of an asset with the return of the same asset during another period of time) and are accompanied by a level of uncertainty which, in this case, we assume as certain. Analytically, since we know the future price is determined by taking sentiment into account, our yield forecast (views) will be determined as:

$$\begin{aligned} \ln \biggl (\frac{S_T}{S_0}\biggr ). \end{aligned}$$

For each stock, we want to be able to modify the optimal composition of a portfolio considering the information obtainable from the market and the prices’ time series. Using the Black–Litterman model, we will calculate the historical average return and the standard deviation, and the stock with the highest Sharpe performance will form the portfolio. Thanks to the Bayesian approach, we can combine historical returns (prior, determined on the basis of historical data) with views, in order to obtain a *posterior* representing the Black–Litterman returns and covariance [51]. These returns *E*(*R*) are determined as:

$$\begin{aligned} E(R) = [(\tau \Sigma )^{-1} + P^{T} \Omega ^{-1}P]^{-1}[(\tau \Sigma )^{-1}\Pi + P^{T} \Omega ^{1}Q], \end{aligned}$$

where *Q* is the vector of views, $\Omega$ is the uncertainty matrix of views, $\Pi$ is the vector of prior expected returns, $\Sigma$ is the covariance matrix, $\tau$ is a scalar constant (defined as weight-on-views) and *P* is the matrix that connects the investor’s views with the model’s assets; while the posterior covariance $\hat{\Sigma }$ is determined as:

$$\begin{aligned} \hat{\Sigma } = [(\tau \Sigma )^{-1} + P^{T} \Omega ^{-1} P]^{-1}. \end{aligned}$$

In the event of market equilibrium, investors would invest in the market portfolio. From this, the determination of the expected returns (prior) takes place through a reverse optimization process of a utility function $(U = w^T \Pi - (\delta /2) w^T \Sigma w)$, to maximize the portfolio return while considering the degree of risk aversion of the investor. The risk aversion factor (market-implied risk premium) can be calculated as the ratio between the excess return of the market portfolio and its variance:

$$\begin{aligned} \delta = \frac{\mu _m - r_f}{\sigma ^2_m}. \end{aligned}$$

The reverse optimization solution to determine the market-implied returns is:

$$\begin{aligned} \Pi = \delta \Sigma w_{mkt}, \end{aligned}$$

where $w_{mkt}$ represents the vector of the weights of each asset in the market portfolio (the market-cap weights).

The key elements of this model are the investor’s views, which are specified in a vector *Q* mapped on the assets in the portfolio via the *P* matrix. The $\Omega$ matrix representing the covariance of the views is not fixed by Black and Litterman in the model description. To solve this problem, it is possible to estimate $\Omega$ using the Idzorek [52] method, according to which the weights in the *P* matrix are proportional to the market capitalization (especially useful for views concerning multiple assets), and the elements of $\Omega$ are set so that the $\omega$ are proportional to the implicit variances of the views. In particular, the variance of a view is calculated as $p_k \Sigma p_k^{'}$, where $p_k$ is a $1\times N$ vector belonging *P* matrix and corresponding to the *k*-th view; and its confidence is determined so that the ratio $\frac{\omega }{\tau }$ is proportional to the variance of the view. Finally, the weights *w* of the assets to invest in the optimal portfolio are given by:

$$\begin{aligned} w = (\delta \Sigma )^{-1} E(R). \end{aligned}$$

### Setting up the machine

The different analyses concerning FinBERT, Monte Carlo and Black and Litterman model were carried out using Python (via Google colab). Thanks to the script released on GitHub by Aract [47], we used pre-trained FinBERT through which we determined polarity and the average sentiment score of different articles. After that, the Monte Carlo method was implemented considering the hourly price of the different stocks (obtained through the *yfinance* package), setting $dt=\frac{1}{45}$ (drift and volatility were determined in the classic way) and generating 10,000 paths starting from $S_0$. Finally, for the Black and Litterman model we used a script^10^ from PyPortfolioOpt in which, however, the views have been “optimized” by obtaining them as previously described.

## Results

Our goal is to exploit the polarity of natural language to improve stock price prediction and subsequent portfolio management. So we can apply the above heuristic to prices and use the yield obtained as a “fine-tuned” view.

### Predicted yield

The sentiment analysis process previously described with FinBERT generated the following results in Table 2.

**Table 2:** Stocks with FinBERT sentiment prediction

Stocks	Polarity	$\gamma$
Microsoft	Positive	0.40
Mondelez	Negative	−0.25
Apple	Positive	0.27
Amazon	Neg./neutral	−0.04
AstraZeneca	Neg./neutral	−0.09
Brembo	Negative	−0.23
Unicredit	Negative	−0.53
EssilorLuxottica	Positive	0.29
UnipolSai	Positive	0.22

The methodology we use to determine the future price is based on simulation. For this purpose, we acquire the historical prices of stocks (for about 2000 hours in the past) to determine the parameters that govern the price process: drift and volatility. However, unlike the everyday use of simulation techniques in which we try to make predictions in the future after days, in this case, we are interested in the **hourly price**. The reason is that the different information that can be acquired in the markets (from newspapers, for example) impacts the price only for a particular limited time.

We can, for example, consider a more extended period from which to extract articles. Let us consider only two stocks (those with the most articles in the Financial Times, belonging to different sectors): Apple and AstraZeneca. The reference period could be from 1/8/2020 to 1/31/2021 to have 88 items for Apple obtaining a $\gamma = 0.37$ and 69 for AstraZeneca, with a $\gamma = 0.29$. These sentiment values are overestimated compared to the one calculated considering only the last article and considering the events of 6 months. If we wanted to use these $\gamma$ values in our heuristic to determine a price after a few hours, we would make a mistake due to the combined effect of the different news. Therefore, overextending this time interval could make the news unusable and frustrate the forecast.

In the first phase, we can consider a forecast interval of 45 hours in the future as a good compromise, starting from the article’s date of publication (indicating with $S_0$ the last price recorded on the day of publication of the article).

The theoretical application of the heuristic described previously consists in determining the drift and volatility parameters from the historical price series and using them to perform a Monte Carlo simulation using $S_0$ as an initial price, generating 10000 paths for each stock.

**Fig. 1:**
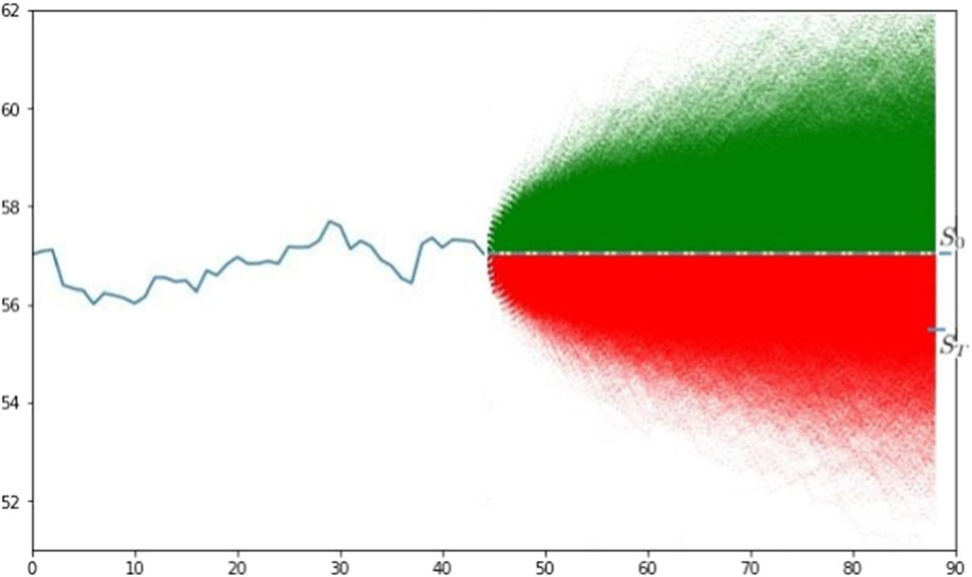
Representation of the Monte Carlo simulation for Mondelez

Figure 1 represents the paths generated with the simulation in the case of the Mondelez stock: the first line represents the 44 hourly prices recorded on the markets; after these hours, we fix $S_0$ and represent it through the dashed horizontal line, which separates the two intervals of the path (based on sentiment, green for positive and red for negative). The predicted price $S_T$ can be determined by applying Eq. 3; in the present case, since the polarity obtained with FinBERT for Mondelez is negative, we will use the red paths. Numerically, since we have all the simulated prices at the last time (the 45*th*), $S_{MC}^{-}$ can be determined simply as the minimum among them; finally, since $\gamma$ is returned directly by FinBERT, we have all the elements for calculating $S_T$. This mechanism was repeated for all stocks whose polarity we determined, obtaining the values shown in Table 3.

**Table 3:** Summary of values used for forecasts and determination of yields

Code	$S_0$	$S_T$	Real pr.	Yield(%)	Real y. (%)	Diff. (%)
MSFT	232.58	**249.10**	242.94	6.63	4.26	2.37
MDLZ	57.02	**55.34**	56.16	−3.03	−1.53	1.5
AAPL	137.08	**145.32**	137.40	5.67	0.23	5.44
AMZN	3239.70	**3216.00**	3330.00	−0.73	2.71	3.44
AZN	52.02	**51.22**	50.36	−1.56	−3.29	1.73
BRE.MI	10.93	**10.42**	10.69	−4.89	−2.24	2.65
UCG.MI	9.094	**8.079**	8.10	−12.56%	−12.27	0.29
EL.MI	121.45	**127.45**	126.30	4.70	3.84	0.86
US.MI	2.176	**2.396**	2.2920	9.18	5.06	4.12

Bold values represent the price predicted with our heuristic

In particular, in the previous table, the $S_0$ column represents the last price recorded on the markets before the article was published. Next, $S_T$ the price predicted with our heuristic; *Real pr.* the price recorded on the markets 45 trading hours (about five days) after $S_0$; the *Yield* column obtained as

4 $$\begin{aligned} Yield = \biggl (\frac{S_T - S_0}{S_T}\biggr ) \times 100 \end{aligned}$$

for each stocks; *Real y.* like the previous column, but based on the actual price recorded

5 $$\begin{aligned} Real \, y. = \biggl (\frac{Real \, pr. - S_0}{Real \, pr.}\biggr ) \times 100 \end{aligned}$$

Finally, *Diff* is the difference (in absolute value) between the two types of yields, the one obtained considering the polarity and the real one.

Some considerations must be made on the results shown in Table 3. Firstly; we can globally evaluate the $\gamma$ score based on the *Diff* column, in the sense that the lower the difference value, the better was the evaluation of the sentences that allowed us to “adjust” the prediction. On average, the difference between the two yields is around 2.48%. The highest deviation concerns the Apple action; this may be due to the presence of further news (released following the article considered) that has changed the polarity. Secondly, almost all signs of the two yields agree, except the Amazon stock. This occurs because the polarity of the article under consideration led us to believe the news to be reasonably neutral when in reality, something happened that pushed the stock higher. These results highlight how the financial sector is heavily impacted in the concise term and that in some cases, 45 hours may be too long.

An important thing to keep in mind is that this method is based on simulation, so the goal is not to identify the exact Borelian (i.e., predict the price that will be recorded) because it would be unrealistic. Instead, the aim is to help the investor/manager transform a feeling expressed in natural language into a numerical value that can be used to manage the portfolio better.

### BL views

At this point, we can use the yield determined operated $S_T$ as a **“fine-tuned” view** to improving the portfolio composition dynamically and over very short periods. With this method, we may be able to “adjust” the composition of a portfolio every time news of a particular type occurs. However, this tool should not be understood as a substitute for human decisions but rather a decision support tool, especially when choosing a view can be tricky based on human sentiment.

We can, for example, consider the previous example portfolio based on *NASDAQ100*. Black–Litterman is used traditionally, with daily historical data for which we will be able to give a forecast in five working days, thanks to the use of FinBERT and hourly prices. To test the effectiveness of the new views, we can consider a positive $\delta$ obtained from a market portfolio. In this case, we used the *NASDAQ100* index (NDX code), which leads to $\delta = 4.4644$. Since only half of the stocks in the portfolio are equipped with new views, we will leave the parameters unchanged for the remainder not considered stocks. We compared two situations:

- The portfolio is managed dynamically through the use of our views (the value of the views corresponds to the value in column Yield of Table 3, summarized in Table 4.
  The confidence value in the view is fundamental since, in this example, we are temporally placed at 1/30/2021, and we calculated the return having no real information on the future (therefore on the Real y.). For this reason, confidence represents our confidence in the sentiment value achieved with FinBERT. In particular, for Amazon, the level of confidence is shallow due to its value close to neutrality;
- We exclude the use of views and consider the portfolio as a whole. In this way, it is possible to see how the $x_k$ values and the overall portfolio performance change.

The overall portfolio performance is measured using the *Sharpe Ratio*. Indicating with $r_P$ and $\sigma _P$ the portfolio return and volatility, respectively, and with $r_f$ the risk-free interest rate (set by default $r_f = 0$), the Sharpe ratio (*SR*) can be defined as:

6 $$\begin{aligned} SR = \frac{r_P-r_f}{\sigma _P}. \end{aligned}$$

Tables 5 and 6 show the prior and posterior returns (obtained by applying the views to the prior), the views used, and the number of stocks that can be purchased considering an initial investment of $100000$ (“No.” column).

**Table 4:** View and confidence for the first case

Stock	View	Confidence
MSFT	0.0663	0.8
MDLZ	−0.0303	0.8
AAPL	0.0567	0.7
AMZN	−0.0073	0.6
AZN	−0.0156	0.8

**Table 5:** Scenario with FinBERT views

Stock	Prior	Posterior	Views	No.
AAPL	0.254144	0.082071	0.0567	185
ADBE	0.219197	0.056681	NaN	27
AMZN	0.251204	0.054660	−0.0073	3
AVGO	0.227665	0.065267	NaN	36
AZN	0.119585	−0.006013	−0.0156	0
MDLZ	0.118381	−0.015487	−0.0303	0
MSFT	0.224680	0.065712	0.0603	75
NFLX	0.231965	0.068042	NaN	28
PEP	0.106722	0.008824	NaN	0
TMUS	0.171236	0.041010	NaN	45

In the polarity-based allocation, there is a leftover of approximately $300$. In Table 6 the views column is missing because they have not been considered (to avoid the presence of a column made up of only NaN.

**Table 6:** Scenario without views

Stock	Prior	Posterior	No.
AAPL	0.254386	0.234386	118
ADBE	0.219155.	0.199155	25
AMZN	0.250988	0.230988	5
AVGO	0.227671	0.207671	27
AZN	0.119690	0.099690	102
MDLZ	0.118377	0.098377	87
MSFT	0.224663	0.204623	56
NFLX	0.231828	0.211828	220
PEP	0.106729	0.086729	28
TMUS	0.172537	0.152537	63

**Fig. 2:**
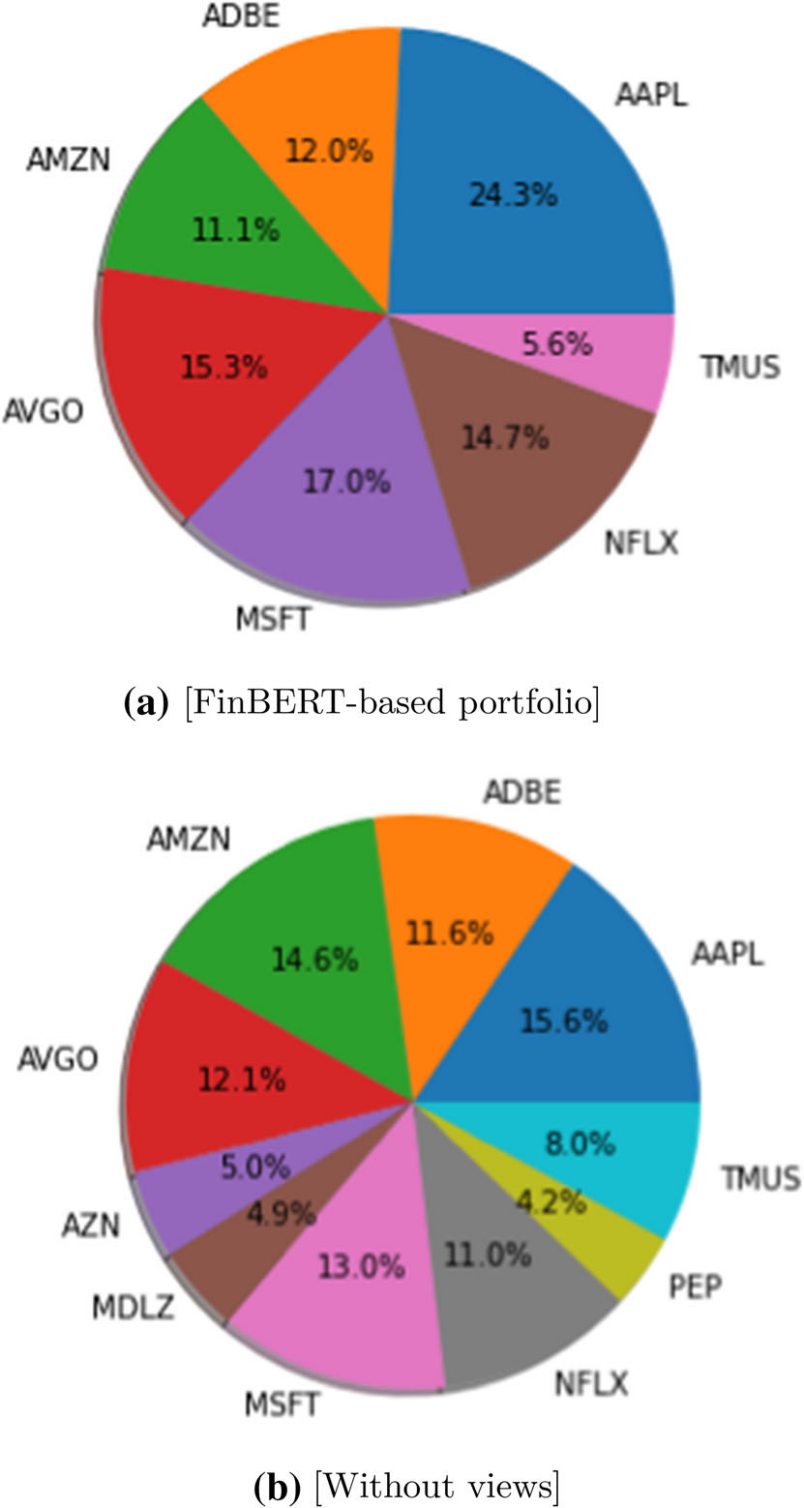
Portfolio composition with and without views

Graphically, in Fig. 2 the combinations of the different stocks in portfolio are represented. In particular, the stocks whose polarity was found to be negative were not taken into consideration to avoid possible losses (for this reason, Fig. 2a shows only seven stocks compared to the ten overall of Fig. 2b).

At this point, we can calculate the portfolio performance based on the prices recorded on 2/5/2021. Then, recalling that the allocation was made on 1/31/2021, we measure the performance of the two previous situations (polarity-based allocation and not) based on future prices, for which the quantity of each stocks is determined on the possible evolution (via heuristics). Table 7 takes up the previous quantities of each stock held in the portfolio (in both cases) and shows the equivalent value of these considering the price blocked at 2/5.

**Table 7:** Equivalence (in $) for the various portfolios

Stock	Price (2/5)	w/FinBERT		Classical	
		No.	Value	No.	Value
AAPL	135.95	185	25150.75	118	16042.10
ADBE	492.12	27	13287.24	25	12303.00
AMZN	3352.14	3	10056.42	5	16760.70
AVGO	449.53	36	16183.08	27	12137.31
AZN	47.62	0	0.00	102	4857.24
MDLZ	55.01	0	0.00	87	4785.87
MSFT	239.69	75	17976.75	56	13422.64
NFLX	550.79	28	15422.12	20	11015.80
PEP	136.06	0	0.00	28	3809.68
TMUS	125.28	45	5637.60	63	7892.64
			103713.96		103026.98

It is already evident from the portfolio value that the allocation made, taking into account the polarity, made it possible to obtain (based on the prices recorded) an increase compared to the portfolio that would have occurred without making any changes. Using Eq. 6, we can calculate the *Sharpe Ratio* for the two portfolios thus constituted, from which we obtain $SR_{FinBERT} = 1.14$ and $SR_{Classical} = 1.07$. This result supports the idea that the interpretation of news obtainable from financial markets through *NLP* models allows improving portfolio management without being influenced by what the news says.

## Conclusions

This paper has tried to improve the Black–Litterman model through sentiment analysis. In particular, our idea is to exploit the sentiment score obtained by applying an NLP model to determine the future price of some stocks based on information that can be found on the markets. Once we obtained the score by applying FinBERT to various articles, we used the Monte Carlo method to discretize the measure present on the paths. This allows us to determine (through a weighted average) an exact future value of the price determined by taking this information into account (i.e., representative of the investor’s inclinations). In this sense, the predicted future price was used to determine a view to be used in a more dynamic version of the Black–Litterman model linked to hourly prices, to allow the composition of a financial portfolio to be modified based on the information on the markets. Through this approach, we can direct the behavior of investors (especially those with less experience) towards a more appropriate choice in the case of news with a particular impact on the markets. This methodology could quantify the impact of news and reassessment the way of operating mainly in the case of negative polarity (which generates fear and could lead to unnecessary purchases/sales). Future work may consider: (i) a BERT model trained in other languages (e.g., Italian) in order to exploit the possible facets of those languages to construct the view as close as possible to the investor’s intention; (ii) longer or shorter time intervals for prices to see how the effectiveness of the application of the Monte Carlo method can change; (iii) use of a greater number of articles for each stock in order to gather a large amount of information about the markets.

## Appendix A comparison with other forecasting techniques

In this appendix, we compare the prediction made with our heuristic and some of the main methods known in the literature. But, first, let’s analyze some statistics of the different datasets used, which we remember being five stocks of the *NASDAQ100* (which belong to the portfolio) and four of the *FTSEMIB*. Table 8 shows the mean, standard deviation, skewness, kurtosis, minimum, and maximum for each dataset. At the same time, the number of elements in the dataset (first column, “No.”) corresponds to the number of hours in the past (starting from the date of publication of the article) that we have considered.

**Table 8:** Main statistics of the time series used

Stocks	No.	Mean	St. Dev	Skewness	Kurtosis	Min.	Max.
MSFT	1156	210.67	10.33	0.19	7.29	181.44	309.42
MDLZ	1170	55.92	2.37	−0.89	−0.30	50.05	63.53
AAPL	1171	113.03	15.34	−0.38	−0.48	79.83	174.31
AMZN	1170	3104.48	223.18	−1.26	1.35	2451.44	3532.39
AZN	1171	53.92	2.21	−0.01	0.60	48.26	66.81
BRE.MI	1350	8.49	0.89	1.14	1.80	6.56	11.30
UCG.MI	1521	7.80	0.76	−0.35	−0.82	6.02	9.40
EL.MI	1137	114.32	4.25	−0.06	−0.81	105.05	124.70
US.MI	1260	2.20	0.13	0.28	−0.60	1.88	2.48

At this point, we can compare our predicted price (after 45 hours) with some of the most popular techniques, and in particular:

- Fischer and Krauss [35] proposed several models such as LSTM, Random Forest (RAF), Deep Neural Network (DNN), and Logistic Regression (LOG) in predicting the performance (returns in their case) of the constituents of the S&P500 index. We can use the previously architectures to make a price prediction (only for future price) and compare it with our obtained value. Table 9 reports the real price recorded, our predicted price (column “Heur”), and the forecast obtained with the various previous models.
  The models of Fischer and Krauss do not consider the sentiment score due to the problem of not being able to transform a single numeric value into a feature, but produce a prediction based only on the training data. We recreated an architecture like theirs (using their hyperparameters), trained it on our data (the different stock datasets defined above with the Open, High, Low, Adj Close and Volume features - OHLCV dataset - acquired by *Yahoo!Finance*^11^), and generated a predicted value after 45 hours. As demonstrated by the authors, the LSTM achieves the best predictive results, followed by the RAF, the DNN, and the LOG. Although they do not consider the value of sentiment, these methods allow us to obtain an excellent prediction. However, with our heuristics and sentiment additions, we are able (in some situations) to align the predicted price to the one recorded better than artificial intelligence techniques.
- Namdari and Li [32] propose using a network based on Multilayer Perceptron (MLP). The main feature of their method is to add to the dataset various features deriving from the technical analysis of the business activity, such as Current Ratio, Inventory Day Sales, Inv/Curr assets, and so on. Unfortunately, we do not have all this information for the different titles we have chosen in our case. However, we will limit ourselves to exploiting a network architecture like the one used by the authors (with the OHLCV dataset) and compare the prediction with our method. Again, in Table 10 we report the real price recorded, our predicted price, and the forecast obtained with the MLP model.
  Also in this case, the use of an MLP network would allow to obtain excellent results but without the possibility of inserting the sentiment score as a feature.

**Table 9:** Prediction comparison with Fischer and Krauss [35] models.

Stocks	Real	Heur.	LSTM	RAF	DNN	LOG
MSFT	242.94	**249.10**	232.58	254.97	227.55	248.75
MDLZ	56.16	**55.34**	57.66	58.98	58.5	59.04
AAPL	137.40	**145.32**	127.16	148.27	133.99	143.76
AMZN	3330.00	**3216.00**	3207.89	3289.32	3177.88	3198.85
AZN	50.36	**51.22**	49.75	59.81	51.62	57.43
BRE.MI	10.69	**10.42**	9.26	10.95	8.75	8.99
UCG.MI	8.10	**8.079**	8.32	7.71	8.45	7.98
EL.MI	126.30	**127.45**	121.44	121.45	119.35	120.67
US.MI	2.2920	**2.396**	2.084	2.169	2.15	2.18

Bold values represent the price predicted with our heuristic

**Table 10:** Prediction comparison with modified Namdari and Li [32] model.

Stocks	Real	Heur.	MLP
MSFT	242.94	**249.10**	246.89
MDLZ	56.16	**55.34**	53.05
AAPL	137.40	**145.32**	134.56
AMZN	3330.00	**3216.00**	3208.12
AZN	50.36	**51.22**	55.75
BRE.MI	10.69	**10.42**	9.06
UCG.MI	8.10	**8.079**	8.34
EL.MI	126.30	**127.45**	120.34
US.MI	2.2920	**2.396**	2.21

Bold values represent the price predicted with our heuristic

## Footnotes

1 Richard Waters in San Francisco, Microsoft shares hit record on work-from-home revenue boost, FT.com, January 27, 2021 Wednesday, available at https://www.ft.com/content/720ba9e9-8cea-4583-b26e-d98a74c4db21.

2 Javier Espinoza in Brussels and Judith Evans in London, EU opens antitrust probe into Cadbury maker Mondelez, FT.com, January 28, 2021 Thursday, available at https://www.ft.com/content/8e449fe8-03c4-416e-b0a0-2c2bc9afa544.

3 Patrick McGee in San Francisco, Apple’s revenue hits another record as it navigates supply chain crunch, FT.com, January 28, 2022 Friday, available at https://www.ft.com/content/25450ffe-e080-4f7a-b8d5-94d23d52af77.

4 Dave Lee in San Francisco, US police and fire departments partnering with Amazon’s Ring passes 2,000, FT.com, January 29, 2021 Friday, available at https://www.ft.com/content/61968b3b-c093-4c4a-a7b7-29b565bc0bc0.

5 Michael Peel, Sam Fleming and Javier Espinoza in Brussels, Brussels publishes EU-AstraZeneca vaccine contract, FT.com, January 29, 2021 Friday, available at https://www.ft.com/content/5a8a87b4-0ea8-4db3-acc4-8a72d3b8b64c.

6 Marco Capponi, Brembo è rimbalzata troppo, Akros abbassa il giudizio a neutral, November 27, 2020, milanofinanza.it, available at www.milanofinanza.it/news/brembo-e-rimbalzata-troppo-akros-abbassa-il-giudizio-a-neutral-202011271058082488.

7 Luca Gualtieri, Unicredit, Mustier getta la spugna, November 30, 2020, milanofinanza.it, available at www.milanofinanza.it/news/unicredit-mustier-getta-la-spugna-202011302118043532.

8 Andrea Montanari, EssilorLuxottica fa shopping in Canada, November 27, 2020, milanofinanza.it, available at www.milanofinanza.it/news/essilorluxottica-fa-shopping-in-canada-202011272011065827.

9 Paola Valentini, UnipolSai batte le attese e conferma gli obiettivi del piano al 2021, November 13, 2020, milanofinanza.it, available at www.milanofinanza.it/news/unipolsai-batte-le-attese-e-conferma-gli-obiettivi-del-piano-al-2021-202011130820313005.

10 https://github.com/robertmartin8/PyPortfolioOpt/blob/master/cookbook/4-Black-Litterman-Allocation.ipynb.
